# Prolonged bacterial carriage and hospital transmission detected by whole genome sequencing surveillance

**DOI:** 10.1017/ash.2024.4

**Published:** 2024-01-30

**Authors:** Alexander J. Sundermann, Marissa P. Griffith, Vatsala Rangachar Srinivasa, Kady Waggle, Graham M. Snyder, Daria Van Tyne, Lora Pless, Lee H. Harrison

**Affiliations:** 1 Microbial Genomic Epidemiology Laboratory, Center for Genomic Epidemiology, University of Pittsburgh, Pittsburgh, PA, USA; 2 Division of Infectious Diseases, University of Pittsburgh School of Medicine, Pittsburgh, PA, USA; 3 Department of Epidemiology, School of Public Health, University of Pittsburgh, Pittsburgh, PA, USA; 4 Department of Infection Control and Hospital Epidemiology, UPMC Presbyterian, Pittsburgh, PA, USA

## Introduction

Healthcare-associated infections (HAIs) may be acquired by transmission in the healthcare setting from common environmental sources or patient-to-patient transmission. In determining possible commonalities, patients may be assumed to have short duration (<90 days) of transmissibility with bacterial pathogens after the initial clinical infection.^1–3^ However, some studies suggest that this assumption may not always hold true, potentially leading to the exclusion of patients as sources of transmission during outbreak investigations.^4–7^ To accurately determine the length of infectiousness and transmission for bacterial pathogens, an ideal study would use serial whole genome sequencing (WGS) to identify genetically related isolates over a prospective period from individual patients.

In November 2016, we began the Enhanced Detection System for Healthcare-associated Transmission in which we systematically performed WGS surveillance for major bacterial pathogens from patients’ clinical infections at our institution to detect, investigate, and interrupt outbreaks.^8–11^ Here, we report incidental findings on patient persistent carriage and potential transmission and their infection prevention implications for outbreak investigations.

## Methods

This study was performed at the University of Pittsburgh Medical Center-Presbyterian Hospital, an adult tertiary care hospital with 758 total beds, 134 critical care beds, and over 400 annual solid organ transplants. Ethics approval was obtained from the University of Pittsburgh Institutional Review Board.

We sequenced select potentially healthcare-associated bacterial pathogens as previously described from November 2016 to August 2019.^10^ In brief, patients’ isolates were collected who were in the hospital for ≥3 days or had a hospitalization in the prior 30 days and sequenced. Duplicate patient isolates were collected if an isolate had not yet been sequenced in the prior 14 days. Isolates were defined as genetically related if they were ≤15 single nucleotide polymorphisms (SNPs) from another isolate except for *Clostridioides difficile* (≤2 SNPs).^10^ Data on patients with duplicate, related isolates were summarized. To identify putative transmission events, we first defined index patients as those with two genetically related isolates identified >100 days apart (arbitrary threshold based upon past literature using 90-day threshold), and then defined exposed patients (with acquisition) as those patients with a genetically related isolate identified between the source patient’s first and last culture date. Transmission events were examined for possible epidemiological routes of transmission (nursing unit, healthcare worker, or procedure).^10^

## Results

The study population includes 4,246 sequenced isolates from November 2016 to August 2019. There were 779 (18.3%) genetically related isolates among 369 unique patients with duplicate isolates (range 2–11 isolates/patient); 3,467 isolates had no genetically related isolates in this data set. The median time from first to last culture date of patients with repeat, related isolates was 33 days (mean 81.9 days) (Figure S1). There were 77 (20.9%) patients with repeat isolates who had isolates related to ≥1 other patient isolate. Of these, 18 (23.4%) patients had >100 days between their first and last isolation of genetically related isolates (range 103–899 days, median 216 days).

Among these 18 index patients, 12 had a clustered isolate to another exposed patient with a culture date between their first and last culture date. Nine of these index/exposed patient pairs had an epidemiological link to another clustered isolate, which included nursing units, shared healthcare workers, shared operating rooms, or shared endoscopes (Table 1). The median time from the index patient’s first culture to the exposure date of the exposed patient was 100 days (range 1–220 days). Culture dates of the linked patients occurred a median of 126 days (range 3–335) after the index patient’s culture date.

**Table 1.** Description of prolonged carriage and transmission events

Cluster	Organism	SNP Distance (Index patient first isolate to exposed patient isolate)	Total Patients in Cluster	Days from index patient culture date to exposed patient date	Days from index patient culture date to exposed patient culture date	Possible exposure
1	*Klebsiella pneumoniae*	6	3	220	335	Nursing Unit
2	*Pseudomonas aeruginosa*	6	7	190	259	Healthcare Worker
3	*Pseudomonas aeruginosa*	1	2	187	201	Operating Room
4	*Pseudomonas aeruginosa*	1	2	178	235	Intensive Care Unit
5	*Pseudomonas aeruginosa*	2	2	100	126	Endoscope
6	*Pseudomonas aeruginosa*	2	2	85	94	Nursing Unit
7	Vancomycin-resistant *Enterococcus faecium*	13	2	28	36	Intensive Care Unit
8	Vancomycin-resistant *Enterococcus faecium*	1	6	27	29	Nursing Unit
9	*Klebsiella pneumoniae*	9	2	1	3	Intensive Care Unit

*SNP: Single nucleotide polymorphism.

## Discussion

In this study, we have demonstrated that patients can have persistent carriage with a genetically related bacterial pathogen for over two years and may contribute to the transmission to other patients during this time. This work highlights the infection prevention implications for patients who may be contributing to ongoing transmission.

The findings of this study further support the consideration of using WGS surveillance rather than reactive WGS. Our prior findings and others show that WGS surveillance has the capability to detect transmission events that would have otherwise been undetected. Here, we show that patients may carry the same bacteria for long durations of time and contribute to transmission, which would not have been considered without the use of WGS surveillance. Healthcare institutions not using WGS surveillance should consider patients with prior infections as potential sources of outbreaks and transmission.

There are limitations to our findings. First, we only sequenced select bacterial pathogens and therefore most likely missed transmission of other pathogens, which suggests that this phenomenon is more common than described in our study. Second, we only sequenced isolates from clinical infections prompted by clinician suspicion, potentially underestimating the duration of colonization and contribution to transmission. Third, we did not assess for patient factors that contributed to the carriage or transmissibility of pathogens. Fourth, these isolates were collected and analyzed retrospectively, and no real-time environmental or screening investigation could be performed to definitely confirm the transmission routes.

In conclusion, we show prolonged carriage of bacteria that can be associated with transmission. Sustained WGS surveillance is likely necessary to completely characterize index and exposed patient relationships. By accurately identifying patients involved in transmission and assessing their potential for persistent carriage, we can develop more effective strategies to prevent and control HAIs in healthcare settings. Combined with prior data, healthcare institutions should consider implementing WGS surveillance approaches for detecting transmission. There is a growing body of evidence on WGS surveillance that shows it can accurately detect outbreaks that are otherwise missed in healthcare settings.^8–12^ Widespread adoption of this approach may ultimately lead to cost savings and better patient outcomes.

## Supporting information

Sundermann et al. supplementary material Sundermann et al. supplementary material

## Supplementary material

For supplementary material accompanying this paper visit http://doi.org/10.1017/ash.2024.4. click here to view supplementary material
