# Supplementary material for: Prolonged bacterial carriage and hospital transmission detected by whole genome sequencing surveillance
Source: Antimicrob Steward Healthc Epidemiol. 2024 Jan 30;4(1):e11. doi: 10.1017/ash.2024.4 (PMC10897709; doi:10.1017/ash.2024.4)
Supplement: Sundermann et al. supplementary material [file S2732494X24000044sup001.docx]

**Figure S1.** Days between same patient first and last isolates, November 2016-August 2019

**
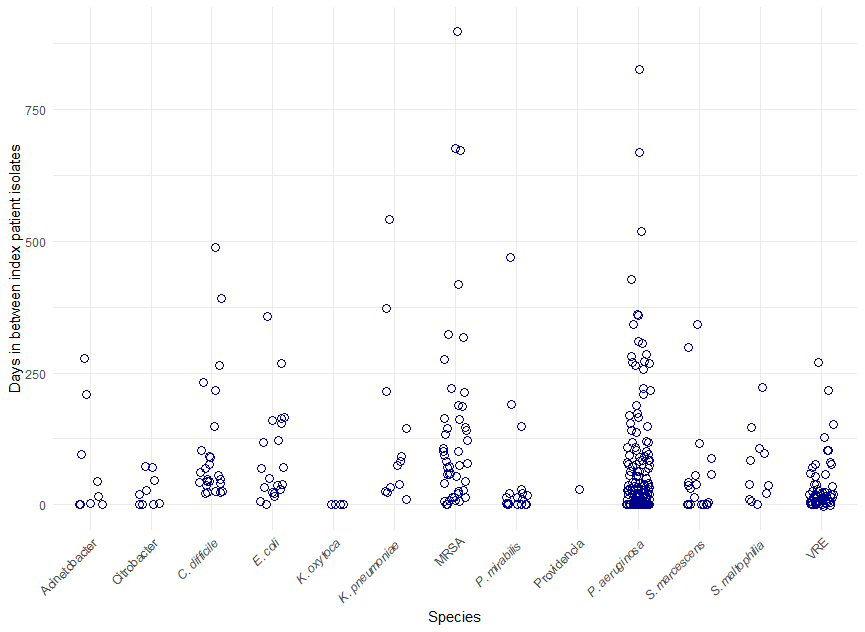
**

*MRSA: Methicillin-resistant *Staphylococcus* *aureus*; VRE: vancomycin-resistant *Enterococcus*
